# Comparison of Content and Quality of Caribbean, International, and High-Income Country-Specific Clinical Guidelines for Managing Type 2 Diabetes Mellitus

**DOI:** 10.3390/ijerph182412868

**Published:** 2021-12-07

**Authors:** Amy Latifah Nixon, Kaushik Chattopadhyay, Jo Leonardi-Bee

**Affiliations:** 1 Division of Epidemiology and Public Health, School of Medicine, University of Nottingham, Nottingham NG7 2RD, UK; amylnixon@outlook.com (A.L.N.); jo.leonardi-bee@nottingham.ac.uk (J.L.-B.); 2 The Nottingham Centre for Evidence-Based Healthcare, A Joanna Briggs Institute Centre of Excellence, University of Nottingham, Nottingham NG7 2RD, UK

**Keywords:** Caribbean, clinical guidelines, high-income countries, improved outcomes, international, management, type 2 diabetes mellitus

## Abstract

Purpose. Type 2 diabetes mellitus (T2DM) is poorly managed in the Caribbean region; therefore, conducting an assessment on the content and quality of clinical guidelines could assist guideline developers in detecting and addressing information gaps. Hence, this study aimed to benchmark and compare the clinical guidelines for T2DM management from the Caribbean to guidelines developed internationally and by high-income countries. Methods. Seven T2DM management clinical guidelines were a priori selected from international and high-income country-specific clinical guidelines and then compared to the country-specific T2DM management clinical guidelines of the Caribbean region. Two reviewers independently assessed content (using a previously piloted data extraction form) and quality using the Appraisal of Guidelines for Research and Evaluation II (AGREE II) tool. Results. The Caribbean clinical guideline was found to contain similar levels of T2DM management topics when compared to international and high-income country-specific clinical guidelines; however, one country-specific clinical guideline from New Zealand was found to have substantially lower levels of content. The clinical guideline from the Caribbean was found to be of low quality and could not be used in practice; however, only three comparator clinical guidelines were found to be of high quality and could be recommended for use in clinical practice. A further three comparator clinical guidelines could be used in practice with minor modifications. Conclusion. Although the T2DM management clinical guidelines from the Caribbean region contained high levels of content with regards to relevant topics, it was of insufficient quality to be used in clinical practice. Therefore, an alternative high-quality clinical guideline, as identified within this study, should be adopted and used within the Caribbean region to manage T2DM until a high-quality region-specific clinical guideline can be developed.

## 1. Introduction

Type 2 diabetes mellitus (T2DM) is a chronic metabolic condition that has major health, social, and economic consequences [1]. Patients with T2DM are known to be at increased risk for microvascular and macrovascular complications (such as diabetic retinopathy, diabetic neuropathy/foot, diabetic nephropathy, coronary heart disease, stroke, and peripheral arterial disease) and even death [2]. Approximately 90% of people with diabetes mellitus have T2DM. Globally, in 2019, approximately 463 million adults were living with T2DM [3]. In 2015, the prevalence of T2DM in the Caribbean region was approximately 9%, and T2DM accounted for about 14% of all deaths [4]. This may be due to the Caribbean’s ethnic makeup, which is predominantly Black or Afro-Caribbean with some people of South Asian descent. These ethnic groups are at significantly higher risks of developing T2DM than compared to other ethnicities [4].

Healthcare practitioners are advised to follow clinical guidelines for managing T2DM, which should contain the best available evidence on how to support and guide both practitioners’ and patients’ decisions on suitable healthcare [5]. Clinical guidelines can improve health outcomes by reducing morbidity and mortality and enhancing quality of life, allowing patients to make informed healthcare decisions, making new procedures and services available to address healthcare issues, and improving the quality of healthcare decisions [6]. Usually, high-quality clinical guidelines reduce differences in clinical practice, encourage the use of effective procedures and services, and eliminate the use of ineffective or less effective procedures and services [6]. Thus, due to the positive impacts that a clinical guideline could have on health outcomes and healthcare, its quality is of great significance. In order to ensure its quality, all steps for developing a clinical guideline should be systematically followed [7].

In the Caribbean region, a national clinical guideline is available for managing diabetes mellitus by primary care doctors, nurses, and allied healthcare professionals [8]. The guideline was first introduced over 25 years ago and, despite the most recent upgrade in 2006, research has shown that the quality of T2DM care and management in the Caribbean has not improved [9]. To date, the content and quality of the clinical guideline have not been robustly evaluated. Therefore, this study aimed to benchmark and compare the content and quality of the Caribbean guideline to international and high-income country-specific clinical guidelines for managing T2DM. Assessing its content will allow for the identification and comparison of information and evidence that support recommendations. Assessing its quality will allow for the evaluation of methodological rigour and transparency of the guideline’s development, and this includes precisely recording and reporting methods and procedures [10]. The issues identified during this research appraisal could be used to improve the clinical guideline.

## 2. Methods

### 2.1. Selection of Clinical Guidelines for Managing T2DM

A priori, a decision was made to compare the existing Caribbean clinical guideline (which is now 15 years old but still in use) [8] to clinical guidelines developed internationally and those from high-income countries. High-income countries usually follow a robust process to develop evidence-based guidelines, which is important for setting a benchmark and for improving healthcare systems. Relevant websites were searched on 29 January 2021 to identify the most recent published versions from the National Institute for Health and Care Excellence (NICE), Scottish Intercollegiate Guidelines Network (SIGN), International Diabetes Federation (IDF), American Diabetes Association (ADA), The Royal Australian College of General Practitioners (RACGP), New Zealand Guidelines Group (NZGD), and Canadian Diabetes Association (CDA). All clinical practice guidelines were required to meet the eligibility criteria of focusing on T2DM management in adults and had to be written in English. Other clinical guidelines were excluded from comparison if they focused on managing specific issues in T2DM (e.g., hyperglycaemia) or if they focused on managing several health conditions together (e.g., prediabetes, diabetes, and cardiovascular diseases), and the extraction of T2DM-specific information was not possible. In short, these were not typical T2DM management guidelines suitable for comparison. We selected one international guideline published by IDF [11] and six high-income country-specific guidelines from Australia [12], Canada [13], New Zealand [14], United States (US) [15], England and Wales [16], and Scotland [17] (see Table 1). Please note that the clinical guideline from Scotland, number 116 [17], also referred to clinical guideline number 154 [18], which provided additional information and, as such, was included as part of clinical guideline number 116 [17].

### 2.2. Comparison of Content of Selected Clinical Guidelines for Managing T2DM

The content of the clinical guidelines was compared based on the following topics by using a previously piloted data extraction form: blood glucose management; body weight assessment and management; blood pressure measurement and management; blood lipids measurement and management; T2DM-associated complications assessment and management; and other healthcare-related issues and advice. Each guideline was read several times to identify topics/subtopics and related recommendations. A full star “
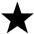
” was assigned to topics/subtopics with adequate information. A half-star “
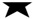
” was assigned to topics/subtopics with limited information. “**NR**” was assigned to topics/subtopics that are not reported in the guideline. Two independent reviewers (AN and GY) were involved in the process, and disagreements were resolved through discussion or with a third reviewer (KC).

### 2.3. Comparison of Quality of Selected Clinical Guidelines for Managing T2DM

The quality of the clinical guidelines was assessed independently by two reviewers (AN and GY) using the Appraisal of Guidelines for Research and Evaluation II (AGREE II) tool, which is a standardised and validated instrument [10]. The AGREE II tool comprises 23 items separated into six domains ((i) scope and purpose; (ii) stakeholder involvement; (iii) rigour of development; (iv) clarity of presentation; (v) applicability; and (vi) editorial independence) and two global rating items ((i) overall quality score and (ii) recommendation for use in practice) [10]. Each item within the AGREE II tool was rated on a seven-point scale (from 1 = strongly disagree to 7 = strongly agree). Average appraisal scores were calculated for each appraiser by using the average rating (1 (strongly disagree) to 7 (strongly agree)) for all items in each domain. Scaled percentages for each domain were then calculated for inter-domain comparison by summing the appraisal ratings of items within a single domain (obtained score), scaling by maximum and minimum possible domain scores, and by converting to a percentage [10]. The overall quality of all included guidelines was summarised and presented using summary statistics (mean). Mean scores of the domains were compared, and the highest and lowest scores were identified. Disagreements in scores were resolved through discussion. Clinical guidelines with a median threshold of ≥70% across all six domains were considered to be of high quality [19].

Two reviewers (ALN and GY) independently scored an overall assessment for each clinical guideline by using a 7-point scale (1 being lowest possible quality to 7 being highest possible quality) together with a statement regarding whether the reviewer recommended the guideline for use (YES, YES with modifications, or NO). Discrepancies between reviewers were discussed and a consensus was reached.

## 3. Results

The Caribbean clinical guideline, last updated in 2006, was developed by the Caribbean Health Research Council (CHRC) and Pan American Health Organization (PAHO) [8] and is between 5 [14] and 14 years [15] older than the other selected guidelines. The clinical guideline encompassed type 1 diabetes mellitus, T2DM, and gestational diabetes in primary care settings. Whilst all of the comparative clinical guidelines focused on primary care as the setting, only three clearly stated the setting (one international clinical guideline, developed by IDF [11] and two country-specific clinical guidelines: one guideline from Canada, developed by CDA, and the other guideline from New Zealand, developed by NZGD [13,14]). Additionally, two country-specific clinical guidelines (Canada, developed by CDA, and New Zealand, developed by NZGD [13,15]) focused on type 1 diabetes mellitus, T2DM, gestational diabetes, and diabetes mellitus in children. A further five comparative clinical guidelines focused solely on T2DM—one international clinical guideline developed by IDF [11] and four country-specific clinical guidelines (Australia, developed by RACGP [12]; Scotland, developed by SIGN [17]; and the joint England and Wales clinical guideline, developed by NICE [16]).

### 3.1. Comparison of Content of Selected Clinical Guidelines for Managing T2DM

The clinical guideline from the Caribbean [8] (developed by CHRC and PAHO) scored well in terms of including a wide range of topics on blood glucose management; body weight assessment and management; blood pressure measurement and management; and T2DM-associated complications assessment and management but generally scored poorly for blood lipid measurement and management and other healthcare-related issues and advice, where limited information was available (Table 2). The clinical guideline from the Caribbean [8] was found to contain similar levels of T2DM management topics compared to six of the comparative clinical guidelines (one international guideline, by IDF [11], and five country-specific guidelines (US ADA [15], Canada CDA [13], England and Wales NICE [16], Australia RACGP [12], and Scotland SIGN [17])). However, for the remaining comparative clinical guideline from New Zealand NZGD [14], the Caribbean clinical guideline [8] was found to possess higher content (28/44 items versus 12/44 items) (Table 2).

### 3.2. Comparison of Quality of Selected Clinical Guidelines for Managing T2DM

Overall, the clinical guidelines only scored more than 70% for two of the six domains: scope and purpose, and clarity of presentation (Table 3). The clinical guideline from the Caribbean [8] scored less than 70% for all domains, where the lowest score of 3% related to the rigour of development and the highest score of 64% related to stakeholder involvement. Domain scores for the seven comparison clinical guidelines demonstrated that three of the clinical guidelines scored 70% or more across all six domains [13,16,17]. A further two of the comparative clinical guidelines (US [15] and Australia [12]) only scored more than 70% in one domain, and another comparative guideline (IDF [11]) scored over 70% in two domains. The remaining comparison clinical guideline from New Zealand scored less than 70% for all domains [14]. The overall quality score for the clinical guidelines ranged from 2 (New Zealand [17]) to the maximum score of 7 (Canada [13], Scotland [17], and England and Wales [16]). The overall quality score for the clinical guideline from the Caribbean [8] was 3, which was the second lowest of all included clinical guidelines. With regards to recommendations for use in clinical practice, the clinical guideline from the Caribbean [8] could not be recommended for use due to its low-quality score; additionally, the clinical guideline from New Zealand [14] was also identified as not recommended for use due to a low-quality score. A further three of the comparison clinical guidelines were recommended for use with modifications (international IDF [11] and two country-specific clinical guidelines from the US ADA and Australia RACGP [12,15]), and the remaining three clinical guidelines were recommended for use without modifications (Canada CDA, England and Wales NICE, and Scotland SIGN [13,16,17]).

## 4. Discussion

This study has identified that the country-specific clinical guideline for managing T2DM developed by CHRC and PAHO for the Caribbean in 2006 [8] contained similar or higher levels of relevant content when compared to the selected international and high-income country-specific T2DM management clinical guidelines; however, the quality of the clinical guideline from the Caribbean [8] was poor and, therefore, cannot be recommended for use in clinical practice. We identified three high-income, country-specific clinical guidelines that were of sufficient quality to be recommended for clinical practice [13,16,17]. We also identified two country-specific clinical guidelines (US and Australia [12,15]) and one international clinical guideline, developed by IDF [11], that could only be recommended for use in clinical practice with modifications. Previous research has highlighted variations in the content of clinical guidelines in relation to managing diabetic neuropathy [20]. In our study, we found that, although the clinical guideline for the Caribbean [8] and several of the comparator clinical guidelines referred to most of the relevant topics, a few subtopics were missing in some of the clinical guidelines, which was a common finding with previous T2DM management guideline appraisal studies [20,21]. The subtopics that were missing from the T2DM management clinical guidelines developed in the Caribbean [8] include triple therapy, bariatric/metabolic surgery, lipid profile, peripheral arterial disease, periodontal disease, cancers, sexual problems in men and women, immunisations for influenza, hepatitis B, pneumonia, bacteraemia and meningitis, older people, fasting (Ramadan), driving, holiday/travel, insurance, and working/shifts. However, even if these subtopics were included in future updates of the clinical guideline, it should be noted that amending the content is not sufficient for being rated as a good quality guideline since the guideline development process plays a vital role [22].

A high-quality clinical guideline can aid in the clinical decision-making process and delivery of high-quality care to T2DM patients in the Caribbean [23]; however, the development of the guideline depends on the availability of resources and a robust development process [24]. Low-quality clinical guidelines can have non-evidence-based, incorrect, contradictory, or not easily identifiable content (and recommendations) [22], thereby impacting healthcare practitioners’ decision making, which can result in significant variations in T2DM management [6]. Thus, low-quality clinical guidelines can result in the use of ineffective interventions, inefficient use of scarce resources, and, most importantly, harm to patients [10,25]. Furthermore, the implementation of clinical guidelines can be challenging due to the influence of a complex set of factors, including political, economic, social, cultural, organisational, and technical factors, and the influence of patients and the public [26,27].

Similar to previous research [21,23,28], we found that two domains, rigour of development and editorial independence, scored poorly for the Caribbean clinical guideline [8] and had mean scores of 50.4% and 39.5%, respectively, across all selected clinical guidelines, thereby highlighting these domains as areas that are generally neglected and require attention. None of the domains in the country-specific clinical guideline from the Caribbean [8] scored highly, resulting in an overall low-quality score. In order to improve the quality of this clinical guideline, an update that ensures editorial independence and follows a rigorous guideline development process is required [7]. For example, a systematic approach was not used to gather or synthesise evidence or to formulate or update the recommendations (e.g., using the Grading of Recommendations Assessment, Development, and Evaluation (GRADE) approach). In addition, the guideline development group should have a greater representation of local T2DM specialists, as this was not adequately addressed before. It should be noted that, although the culture within the Caribbean is similar, other important issues, such as the economic status of individual countries, are different.

Although the Caribbean clinical guideline [8] was first produced in 1995, the last update occurred more than a decade ago in 2006, thus increasing the potential for the guidance to poorly reflect current best practices. This clinical guideline [8] was originally developed based on the economic situation, culture, and healthcare systems in the Caribbean region and was designed to improve patient care and reduce morbidity and mortality by effective management of diabetes. Since this guideline was updated [8], there have been significant advancements in the field of diabetes and T2DM management, such as new and effective medicines including glucagon-like peptide-1 (GLP-1) receptor agonists as well as its alternatives; individualised care regimes tailored to the patient’s health status; prediction models; and recommendations to change current therapeutic practices made by the American College of Physicians [16,29,30,31,32,33], therefore resulting in potential inadequacies and inconsistencies in the guideline. This implies that healthcare professionals in the Caribbean region are not receiving the appropriate clinical guidance needed to ensure the provision of adequate care for patients’ management of T2DM, which could result in poor quality healthcare and poor health outcomes (high mortality and morbidity rate).

In order to improve the clinical guideline in the future and, ultimately, to improve the overall quality of primary, secondary, and tertiary care, and individual outcomes in T2DM, a change from reactive to predictive, preventive, and personalised medicine is required [33,34,35]. For example, models, such as the nomogram, can help in identifying and predicting persons who are at risk of T2DM more efficiently as well as allow persons to develop strategies to combat or prevent the onset of T2DM [33]. The use of personalised medicine in ensuring that a person’s genetic makeup can be used to develop approaches for treating and monitoring T2DM should also be considered [33,34].

A strength of this study is that a recognised and validated tool was used to assess the quality of the clinical guidelines. The AGREE II tool was developed to address variability in guideline quality [10,19,21,23,36] and is recommended by the World Health Organization (WHO), the Guidelines International Network (GIN), and the Council of Europe for its reliability in appraising clinical guidelines [37]. We decided to compare the existing Caribbean clinical guideline with clinical guidelines from high-income countries as the latter would set a benchmark. In contrast, clinical guidelines from low-income and middle-income countries (LMICs) would not have served the purpose of setting the benchmark due to multiple issues, including approaches used to develop the guidelines. In addition, it would have been difficult to select one LMIC clinical guideline over another for comparison and to justify the selection. Finally, in many LMICs, such clinical guidelines are not available. Clinical guidelines written in English were included, and the selection of clinical guidelines was not systematic, which could have resulted in a biased sample of guidelines; however, we attempted to overcome this bias by choosing a range of international and high-income country-specific guidelines published by leading societies, organisations, or associations. Although the Caribbean comprises mainly LMICs and, therefore, access to funding and resources for developing a clinical guideline is likely reduced compared to high-income countries, it was deemed important that comparisons were made with such leading societies, organisations, or associations in order to reflect potential gold standards and minimise the potential for inadvertently identifying low-quality clinical guidelines. Another limitation of this study was that only clinical guidelines published in the English language were considered, thereby potentially introducing English language bias.

## 5. Conclusions

The clinical guideline developed and used within the Caribbean region for managing T2DM was found to contain high level content with regards to relevant topics but was of insufficient quality to be used in clinical practice. Therefore, it is recommended that the existing high-quality clinical guideline as identified within this study should be adopted and used for the clinical management of T2DM within the Caribbean region until a high-quality region-specific guideline is developed.

## Figures and Tables

**Table 1:** Outline of selected clinical guidelines for managing T2DM.

	Publishing Societies/Organisations/Associations	Geography of the Guideline	Name of the Guideline	Last Updated
1	Caribbean Health Research Council (CHRC) and Pan American Health Organization (PAHO) [8]	Country-specific (Caribbean)	Managing diabetes in primary care in the Caribbean	2006
2	International Diabetes Federation (IDF) [11]	International	Recommendations for managing type 2 diabetes in primary care	2017
3	The Royal Australian College of General Practitioners (RACGP) [12]	Country-specific (Australia)	General practice management of type 2 diabetes	2016
4	Diabetes Canada and Canadian Diabetes Association (CDA) [13]	Country-specific (Canada)	Diabetes Canada 2018 clinical practice guidelines for the prevention and management of diabetes in Canada	2018
5	New Zealand Guidelines Group (NZGD) [14]	Country-specific (New Zealand)	Guidance on the management of type 2 diabetes 2011	2011
6	American Diabetes Association (ADA) [15]	Country-specific (United States)	Standards of medical care in diabetes	2020
7	National Institute for Health and Care Excellence (NICE) [16]	Country-specific (England and Wales)	Type 2 diabetes in adults: management	2020
8	Scottish Intercollegiate Guidelines Network (SIGN) [17]	Country-specific (Scotland)	Management of diabetes: a national clinical guideline	2017

**Table 2:** Comparison of content of selected T2DM management clinical guidelines.

T2DM Management		Guidelines							
		Caribbean (CHRC/PAHO) [8]	International (IDF) [11]	Australia (RACGP) [12]	Canada (Diabetes Canada/CDA) [13]	New Zealand (NZGD) [14]	United States of America (ADA) [15]	England (NICE) [16]	Scotland (SIGN) [17]
**T2DM diagnosis**	T2DM diagnosis	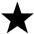	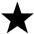	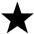	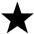	**NR**	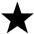	**NR**	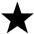
**Blood glucose management**	T2DM self-management education	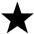	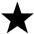	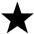	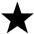	**NR**	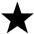	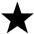	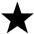
	Self-monitoring of blood glucose	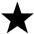	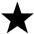	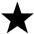	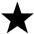	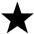	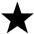	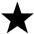	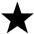
	Blood glucose targets	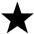	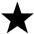	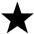	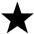	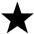	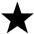	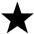	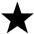
	Healthy diet	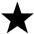	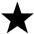	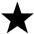	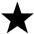	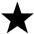	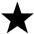	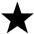	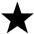
	Medical nutrition therapy (tailored diet) ^a^	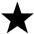	**NR**	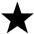	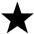	**NR**	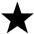	**NR**	**NR**
	Physical activity	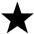	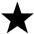	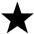	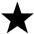	**NR**	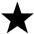	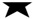	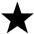
	Smoking cessation	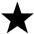	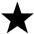	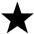	**NR**	**NR**	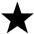	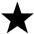	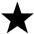
	Reduction in alcohol consumption	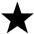	**NR**	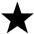	**NR**	**NR**	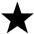	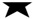	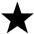
	Initial pharmacological treatment- Monotherapy (one oral drug) ^b^	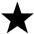	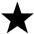	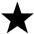	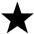	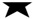	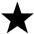	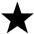	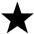
	Dual therapy (combination therapy, including oral drugs and insulin) ^c^	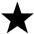	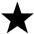	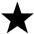	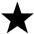	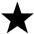	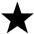	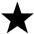	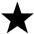
	Triple therapy (combination therapy, including oral drugs and insulin) ^d^	**NR**	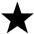	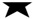	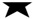	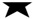	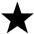	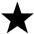	**NR**
	Insulin therapy (insulin only)	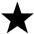	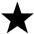	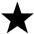	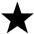	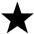	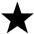	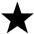	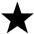
**Bodyweight assessment and management**	Body mass index (BMI) assessment	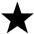	**NR**	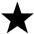	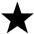	**NR**	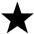	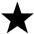	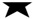
	Waist circumference	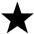	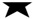	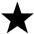	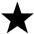	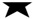	**NR**	**NR**	**NR**
	Anti-obesity drugs ^e^	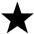	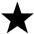	**NR**	**NR**	**NR**	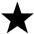	**NR**	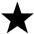
	Bariatric/metabolic surgery ^f^	**NR**	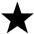	**NR**	**NR**	**NR**	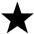	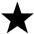	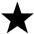
**Blood pressure measurement and management**	Blood pressure checks and targets	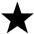	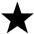	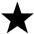	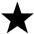	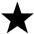	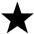	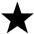	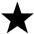
	Antihypertensive treatment	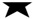	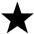	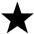	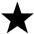	**NR**	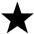	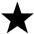	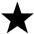
**Blood lipids measurement and management**	Lipid profile	**NR**	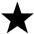	**NR**	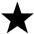	**NR**	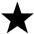	**NR**	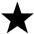
	Lipid modification therapy (e.g., statin)	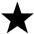	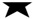	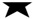	**NR**	**NR**	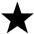	**NR**	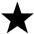
**T2DM associated complications (and conditions) assessment and management**	Hypoglycemia	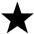	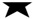	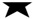	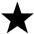	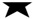	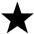	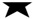	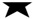
	Diabetic ketoacidosis	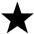	**NR**	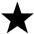	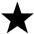	**NR**	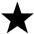	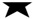	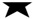
	Diabetic retinopathy	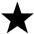	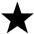	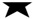	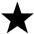	**NR**	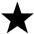	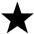	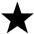
	Diabetic neuropathy	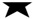	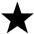	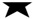	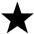	**NR**	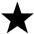	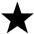	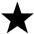
	Diabetic nephropathy	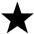	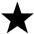	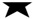	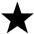	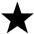	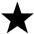	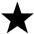	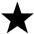
	Cardiovascular disease	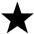	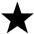	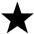	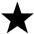	**NR**	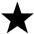	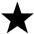	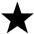
	Antiplatelet treatment (e.g., aspirin for cardiovascular disease)	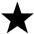	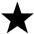	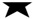	**NR**	**NR**	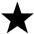	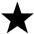	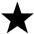
	Peripheral arterial disease	**NR**	**NR**	**NR**	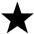	**NR**	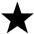	**NR**	**NR**
	Diabetic foot (foot care)	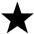	**NR**	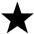	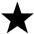	**NR**	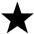	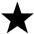	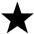
	Periodontal disease	**NR**	**NR**	**NR**	**NR**	**NR**	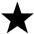	**NR**	**NR**
	Cancers	**NR**	**NR**	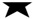	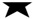	**NR**	**NR**	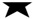	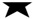
	Sexual problems in men and women ^g^	**NR**	**NR**	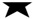	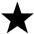	**NR**	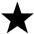	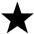	**NR**
	Mental health (including depression and anxiety)	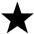	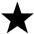	**NR**	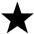	**NR**	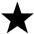	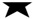	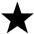
**Other healthcare-related issues and advice**	Immunisations for influenza, hepatitis B, pneumonia, bacteraemia, and meningitis	**NR**	**NR**	**NR**	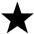	**NR**	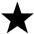	**NR**	**NR**
	Skin examination	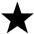	**NR**	**NR**	**NR**	**NR**	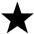	**NR**	**NR**
	Older people	**NR**	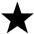	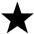	**NR**	**NR**	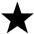	**NR**	**NR**
	Referral to other specialists for advice or treatment	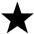	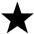	**NR**	**NR**	**NR**	**NR**	**NR**	**NR**
	Fasting, including during religious/socio-cultural festivals (e.g., Ramadan)	**NR**	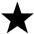	**NR**	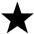	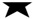	**NR**	**NR**	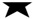
	Driving	**NR**	**NR**	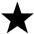	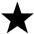	**NR**	**NR**	**NR**	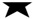
	Holiday/travel	**NR**	**NR**	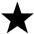	**NR**	**NR**	**NR**	**NR**	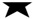
	Insurance	**NR**	**NR**	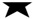	**NR**	**NR**	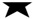	**NR**	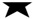
	Working/shifts	**NR**	**NR**	**NR**	**NR**	**NR**	**NR**	**NR**	**NR**
	Principles of providing care	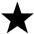	**NR**	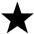	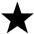	**NR**	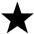	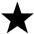	**NR**

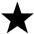
 Found as a heading or subheading in the guideline. 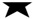 Limited information (i.e., briefly mentioned in the guideline but not as a heading or subheading). **NR**—not reported in the guideline. ^a^ Medical nutrition therapy is a therapeutic approach for treating T2DM and its symptoms by using a specifically tailored diet devised and monitored by a medical doctor or registered dietitian/nutritionist. ^b^ Monotherapy is the initial treatment regimen with one oral drug, usually metformin. ^c^ Dual therapy is the addition of a second drug to a regimen in which one drug for T2DM is not managing a person’s blood glucose. ^d^ Triple therapy is the addition of a third drug to a regimen in which two drugs for T2DM are not managing a person’s blood glucose. ^e^ Anti-obesity drugs are also known as weight loss medications that reduce or control body weight. ^f^ Bariatric/metabolic surgery is a weight loss surgery that is used as a treatment for people who are very obese and to reduce risk for other diseases, such as metabolic or cardiovascular disease complications. ^g^ Sexual dysfunction in men and women includes low testosterone in men and vaginal dryness in women, respectively.

**Table 3:** Comparison of quality of selected T2DM management clinical guidelines.

Guidelines	Domains				Overall Guideline Assessment			
	Scope and Purpose	Stakeholder Involvement	Rigour of Development	Clarity of Presentation	Applicability	Editorial Independence	Overall Quality Score	Recommended for Use in Practice
Caribbean (CHRC/PAHO) [8]	58%	64%	3%	58%	38%	50%	3	No
International (IDF) [11]	100%	61%	23%	94%	35%	4%	4	Yes, with modifications
Australia (RACGP) [12]	25%	36%	35%	100%	58%	25%	4	Yes, with modifications
Canada (Diabetes Canada/CDA) [13]	94%	94%	85%	100%	73%	71%	6	Yes
New Zealand (NZGD) [14]	25%	42%	16%	58%	31%	25%	2	No
United States of America (ADA) [15]	69%	64%	51%	100%	65%	46%	5	Yes, with modifications
England (NICE) [16]	100%	92%	99%	97%	92%	67%	7	Yes
Scotland (SIGN) [17]	100%	100%	91%	97%	92%	92%	7	Yes
Mean	71.4	69.1	50.4	88	60.5	39.5		
